# A Platform for Combined DNA and Protein Microarrays Based on Total Internal Reflection Fluorescence

**DOI:** 10.3390/s120201800

**Published:** 2012-02-09

**Authors:** Alexander Asanov, Angélica Zepeda, Luis Vaca

**Affiliations:** 1 TIRF Technologies, 951 Aviation Parkway, Suite 700, Morrisville, NC 27560, USA; 2 Instituto de Investigaciones Biomédicas, Universidad Nacional Autónoma de México, Ciudad Universitaria, DF 04510, México; 3 Instituto de Fisiología Celular, Universidad Nacional Autónoma de México, Ciudad Universitaria, DF 04510, México; E-Mail: lvaca@ifc.unam.mx

**Keywords:** TIRF, molecular beacon (MB), point-of-care diagnostics (POCD), protein microarrays, DNA microarrays

## Abstract

We have developed a novel microarray technology based on total internal reflection fluorescence (TIRF) in combination with DNA and protein bioassays immobilized at the TIRF surface. Unlike conventional microarrays that exhibit reduced signal-to-background ratio, require several stages of incubation, rinsing and stringency control, and measure only end-point results, our TIRF microarray technology provides several orders of magnitude better signal-to-background ratio, performs analysis rapidly in one step, and measures the entire course of association and dissociation kinetics between target DNA and protein molecules and the bioassays. In many practical cases detection of only DNA or protein markers alone does not provide the necessary accuracy for diagnosing a disease or detecting a pathogen. Here we describe TIRF microarrays that detect DNA and protein markers simultaneously, which reduces the probabilities of false responses. Supersensitive and multiplexed TIRF DNA and protein microarray technology may provide a platform for accurate diagnosis or enhanced research studies. Our TIRF microarray system can be mounted on upright or inverted microscopes or interfaced directly with CCD cameras equipped with a single objective, facilitating the development of portable devices. As proof-of-concept we applied TIRF microarrays for detecting molecular markers from *Bacillus anthracis*, the pathogen responsible for anthrax.

## 1. Introduction

The demands of various biological studies, including gene profiling, drug screening, and clinical diagnostics has stimulated the development of relatively simple, reliable, and high throughput methods allowing the detection of multiple DNA targets in a small volume of biological sample. Advanced detection technologies have been developed based on various analytical techniques such as fluorescence [1–4], surface plasmon resonance (SPR) [5], quartz crystal microbalance [6], and field effect transistors [7]. Among these DNA detection methods, fluorescence-based assays are currently the most popular techniques for multiplexed DNA detection [8,9]. Surface-enhanced Raman scattering (SERS) has also been considered as a promising method for multiplexed DNA detection [10,11] because of its molecular specificity [10,11] and insensitivity to quenching [12]. These advantages have led to the development of a number of SERS sensing platforms [13–15]. However, the reproducibility of SERS signals and ability to detect multiple target molecules in very small sample volumes is still a challenge for practical multiplexed SERS sensors.

DNA microarray is a multiplex technology used in molecular biology and medicine to identify DNA fragments expressed in an organism or tissue [16]. It consists of an ordered series array of thousands of microscopic spots of DNA oligonucleotides (known as probes), each containing a few picomoles of different probes printed on top of silica slides [16]. Like DNA, protein arrays contain thousands of microspots, each of which selectively binds target proteins found in samples. In conventional microarrays, the probes are attached via surface engineering of the silica slides, which may involve covalent modifications to facilitate the adhesion of the probes to the glass surface. The sample is labeled using fluorescent molecules, which are later used to identify sample association to the probe. Typical microarray spots are 100 microns in diameter allowing the printing of thousands of spots in high-density arrays [17,18]. More recent (less conventional) methods of labeling samples may include the use of quantum dots, with the added advantage of being resilient to photo-damage [19]. Another relevant enhancement to the microarray technology is the recent introduction of molecular beacons, a technology we will use extensively during the present study. The use of molecular beacons eliminates the need for labeling the sample, an advantage we will discuss in greater detail later on.

Thus, microarray is a high throughput technique, which facilitates the screening of entire transcriptomes using a single slide. Protein microarrays also detect multiple protein targets in parallel [17,20]. Simultaneous detection of nucleic acids (NA) such as DNA/RNA and protein markers is necessary and advantageous for numerous biomedical applications. However, the temperature and buffer composition for traditional NA and protein microarrays are incompatible for combined detection experiments. Combining NA and protein detection in single system is a challenging task [21].

In the present study we overcame this challenge and developed a novel combined TIRF-based microarray platform that detect NA and protein in the assay, at the same surface of TIRF sensor chip, in a single flow chamber with the same buffer and at room temperature.

This technology provides rapid results (in seconds) and renders association and dissociation kinetics in real time. We have named this technology LG-TIRF-MB. The use of TIRF ensures surface selectivity and enhanced signal-to-noise ratios, efficiently rejecting auto fluorescence from samples and background [22].

As proof-of-concept we use LG-TIRF-MB to identify DNA from *Bacillus anthracis*, the pathogen responsible for anthrax. Anthrax is an acute disease that afflicts humans. Most forms of the disease are lethal if not treated promptly. Anthrax has gain attention recently not only because is endemic in several regions of the world, but also because it has been used as a weapon in bioterrorism in recent years. We demonstrate also the parallel identification of model protein markers, using the same microarray slide.

Furthermore, we show that studying the association and dissociation kinetics allows for the identification of single nucleotide mismatches in the target DNA. TIRF microarrays in conjunction with reagentless molecular beacons assays require minimum sample preparation and no labeling, facilitating the use of scarce samples, which is always a limiting factor in molecular diagnostics.

## 2. Experimental Section

### 2.1. Reagents and Materials

Synthesized and purified molecular beacons, complimentary sequences, mismatched and non-complimentary DNA oligomers were purchased from Integrated DNA Technologies (IDT, Coralville, IA, USA). Biotinylated BSA, Sigmacote solution, all buffer components were purchased from Sigma-Aldrich (St. Louis, MO, USA), Streptavidin was purchased from Jackson Immunoresearch (West Grove, PA, USA). Hybridization buffer (PBST) containing 50 mM TRIS-HCl (pH 8.0), 5 mM KCl, 100 mM NaCl, 5 mM MgCl_2_ (pH 7.4) was used in all experiments reported. The reconstitution buffer contained in addition 4M urea. The phosphate buffered saline (PBS) contained NaCl (137 mM), KCl (2.7 mM), NaHPO (10 mM), KHHPO (2 mM), CaCl_2_, (1.8 mM, pH 7.4).

### 2.2. DNA Probe Design

The molecular beacon (MB1) was designed to target *pag* (protective antigen gene) residing on xo1 plasmid of *B. antracis* (Table 1). The loop sequence and one side of the stem is complimentary to 24 nucleotide fragment of *pag* gene in the region of diagnostic primer PA8 recommended for identification of *B. antracis* by the World Health Organization (WHO). Molecular beacons MB2 was designed to identify a different region form the target *pag.* MB2 was used to demonstrate the multiplexing capacity and improved accuracy of the LG-TIRFM-MB system in combination with TIRF microarrays. The loop from MB2 compliments to 24 nucleotides (1,481–1,505) from the target *pag.*

The molecular beacon was equipped with HEX as the fluorophore-reporter dye, BHQ1 as the fluorescence quencher, and a biotinylated thymidine base on the 3′- stem proximal to the quencher. This biotin-modified thymidine facilitated the immobilization of the beacon to the surface glass.

The complimentary and mismatched DNA probes were designed so that six bases at the 3′ are complimentary to the 5′- stem of the MB as it was shown to improve hybridization (Table 1).

### 2.3. Sensor Surface Preparation for MB and Antibody Immobilization

UV silica TIRF slides 25.4 mm × 76.2 mm × 1.0 mm (TIRF Technologies, Inc., Morrisville, NC, USA) were cleaned in chromic acid heated to 70 °C for 20 min, then rinsed with distilled water and air-dried. The slides were then treated with Sigmacote (Sigma, St. Louis, MO, USA) to achieve uniform hydrophobicity. Slides were incubated with 0.1mg/mL biotinylated BSA in PBS pH 7.4 to allow for adsorption of BSA to the hydrophobic surface. The slides then were rinsed extensively with PBS. After rinsing, the slides with irreversibly adsorbed biotinylated BSA were incubated with 0.1 mg/mL streptavidin for 4 min to allow binding to biotin, following by rinsing with PBS. After these procedures, the slides were ready for printing and contained high density of biotin-binding sites because each streptavidin molecule contains four biotin-binding domains.

### 2.4. Printing MB and Antibody Microarrays

Molecular beacons (MB) were printed on the surface of previously prepared microarray slides (as described above). MB were synthesized with a biotin pendant to allow association to streptavidin present on the surface of the previously prepared slides.

For antibody printing, we utilized two antibodies. Immunopure™ biotinylated mouse (antibody 1) and rabbit (antibody 2) anti-human IgG were purchased from ThermoScientific (cat# 31784 and 31786, respectively), and reconstituted to 1.5 mg/mL according to the manufacturer’s guidelines and stored at 4 °C. For antibody microarray studies, two secondary antibodies were utilized. Alexa Fluor 532-labeled goat anti-mouse and goat anti-rabbit IgG antibodies were obtained from Invitrogen (cat# A11002 and A11009, respectively), they were reconstituted to 2 mg/mL, and stored at 4 °C according to the supplier’s instructions.

Printing of both, MB and antibodies was carried out using 380 micrometer pins attached to the BioOdyssey microarray printer (BioRad, Hercules, CA, USA). The temperature and humidity conditions inside the printing chamber were 25–30 °C and 60–65%, respectively. MB were printed at a final concentration of 0.3 μM and for antibody printing the primary antibody was used at a concentration of 100 μg/mL.

Post printing, the slides were briefly rinsed with 2 × 200 uL of SuperStreptavidin Blocking Buffer (ArrayIt, Sunnyvale, CA, USA), washed by gentle rocking in 2 × 3 mL PBST (10–30 min each) in a polystyrene Petri dish, rinsed with plain PBS and stored dry at 4 °C in a 50-mL disposable centrifuge tube until further use.

### 2.5. TIRF Instrumentation

The LG-TIRF-MB system was designed by TIRF Technologies Technologies, Inc. in collaboration with Angélica Zepeda and Luis Vaca. The system was mounted on top of an inverted Nikon microscope equipped with a 2× objective. Excitation wavelength was 532 nm using a 150 mW laser and emission intensity was collected at 554 nm. An iXon electron multiplied cooled (EM-CCD) camera (Andor Technology) was utilized for image acquisition. The camera was cooled down to −80 °C prior to beginning acquisition. Time-based acquisitions were obtained with time intervals of 1 second and integration time of 0.5 seconds. Typical experiments were carried out for 12–15 minutes and contained hundreds of microarray images.

### 2.6. Experimental Procedures and Conditions

Every experiment consisted of the following steps controlled by the SmartFlow digital fluidics system (TIRF Technologies, Inc.):

1. Time-based acquisition of fluorescence signal was started. The background signal was collected for 60–80 s.
2. The solution of complementary, mismatched or non-complementary DNA (500 nM in hybridization buffer, total volume of 900 μL) was introduced into the flow cell of the LG-TIRF-MB system using a rapid injection protocol (flow rate was 70 μL/s). During complementary DNA application the system acquired continuously images of fluorescence signal from the arrays at the TIRF surface.
3. Pure hybridization buffer (PBST) was dispensed throughout the flow cell for 100 s (flow rate 12.5 μL/s) to remove non-hybridized sample.
4. When studying combined DNA-protein microarrays, the secondary antibody conjugated to Alexa 532 was applied to the chamber in PBST at a flow rate of 70 μL/s, at a final dilution of 5,000×.
5. For microarray reconstitution (regeneration), the solution contained 4 M urea (Experimental Section). The solution was applied during image acquisition at a rate of 70 μL/s. The flow was interrupted after fluorescence reached background levels, indicating the microarray was fully reconstituted and ready for the next cycle of sample application.

## 3. Results and Discussion

### 3.1. Design of the LG-TIRF-MB System

**Figure 1.**
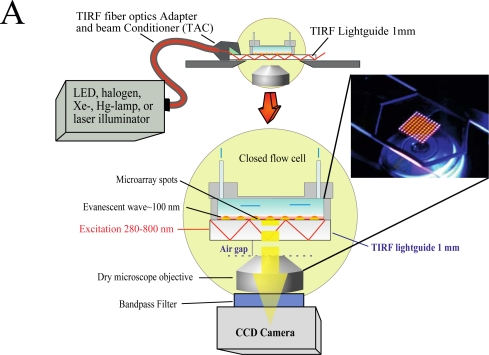

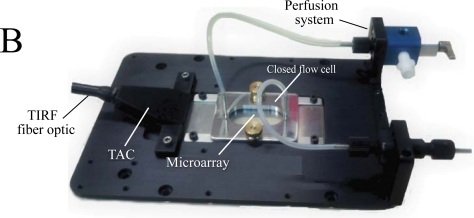
Implementation of the LG-TIRF-MB system. (**A**) Illustration of the different components forming the LG-TIRFM-MB system. Any light source coupled to the mm fiber optic is directed to the beam conditioner (TAC) resulting in a change from “spot-geometry” into “line geometry” of the excitation beam at the TAC outlet. Light sources include light emitting diodes (LEDs), halogen, mercury lamps or lasers. The fiber optic bundle at the end of the TAC couples the excitation light to the slide, which carries DNA-protein microarray at its upper surface. Light then travels bouncing up and down the facet of the slide. At the top of the slide, the light reflecting at the silica/water interface at angles larger than critical produces the evanescent wave. The closed chamber where the microarray is contained facilitates injection of sample bioanalyte solutions and washing procedures. At the bottom of the system is located the low magnification objective (typically 2× or less) and an emission filter. An EM-CCD camera is attached to the microscopy port. The photograph (inset) shows a picture of one microarray slide above the objective. (**B**) Photograph of the actual system showing the tubing used for perfusion, fiber optic bundle, TAC and microarray slide mounted in the (hermetically sealed) closed chamber.

We have developed an add-on system (Figure 1) which can be mounted on top of any inverted microscope and use virtually any light source to produce the evanescent wave (EW) needed for total internal reflection fluorescence (TIRF).

The use of low magnification (2×) objectives facilitates the visualization of the entire microarray. In the present study we used silica slides, but the system is capable of using conventional microarray glass slide (1 mm thick) as light-guide to produce and disperse the evanescent wave (EW). The system is factory-aligned and the incident angle is fixed to a value larger than critical, ensuring reproducible illumination of the microarray in total internal reflection mode. The system is equipped with a closed flow cell to facilitate the rapid addition of samples and rinsing procedures. Since we use the microarray slide as a TIRF lightguide and molecular beacons as reagentless assays, we have named the system Lightguide-based TIRF (LG-TIRF-MB). We have described the technical details of light-guided TIRF elsewhere [23].

Because the EW decays exponentially with the distance from the slide surface, only fluorophores located at or near the surface of the slide are excited and fluoresce [24]. Thus, TIRF is a surface selective technique, which renders information only from a shallow (submicron) area near the slide (typically less than 100 nm) [25]. Surface selectivity prevents auto fluorescence commonly associated with biological samples, while enhancing signal-to-noise ratios, both important elements when considering point-of-care diagnostic (POCD) systems.

The LG-TIRF-MB system consists of fiber optics adapter and beam conditioner (TAC) and a slide-lightguide (Figure 1). TAC is a custom designed fiber optics bundle, which converts “spot-geometry” of the excitation beam from any illuminator (at the inlet of TAC) into “line geometry” of the excitation beam at the TAC outlet. This part of the system is essential to ensure even distribution of the EW along the slide/lightguide. Light propagates through the microarray slide entering the TIRF slide line-end of TAC via the thin edge (1 mm × 25.4 mm) of the rectangular slide (25.4 mm × 76.2 mm × 1 mm). Approximately 90% of the excitation beam reflects from the top and bottom surfaces of the slides at angles larger than critical (for water/glass interfaces about 61.7°). The beam is approximately 16 mm wide at the center of a 25.4 mm × 76.2 mm slide. This provides a sufficiently wide and uniform EW to excite all the spots on the microarray simultaneously. A CCD camera is coupled to the microscope to collect the fluorescent signal from the microarray.

### 3.2. A Robust and Reusable Microarray System

DNA-based molecular beacons (MB) and antibodies were printed as low-density arrays on the surface of silica microscopy slides (Material and Methods, see also supplementary material Figure S1). The diameter of each spot was approximately 400 microns, and the pitch 600 microns. At this density several dozen printed spots could be visualized simultaneously with our LG-TIRF-MB system using a conventional 2× objective.

Conventional microarray technology includes incubation steps in which the sample is heated to facilitate the association to the probe printed on the microarray glass surface. The LG-TIRF-MB technology is designed to facilitate the association between target and MB occurring at room temperature. In order for target-MB association to occur at room temperature, molecular beacons must be designed paying special attention to the stem and length of the MB loop and stem complementarity, according to previously published rules [25].

**Figure 2.**
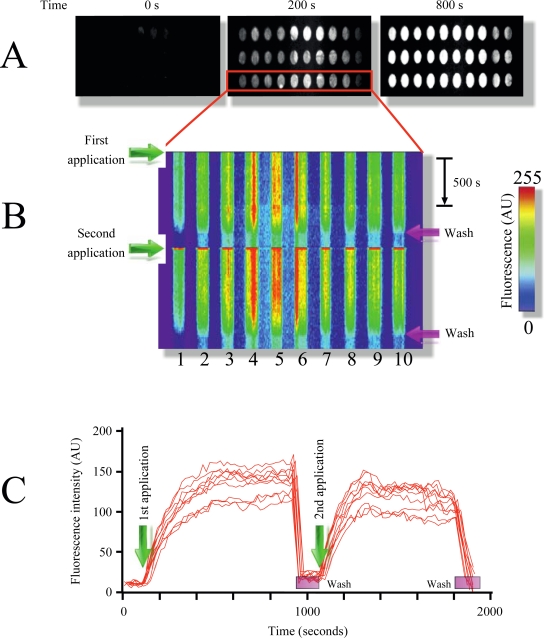
Typical microarray experiment using the LG-TIRF-MB system. (**A**) Picture of the viewing area accessible to the 2x objective (the image shows 30 spots). (**B**) Time course of the microarray activation upon DNA sample addition. Notice the reproducibility of the response when all spots show increments in fluorescence with similar time courses. The fluorescence was plotted X–Y and time to illustrate the entire experiment in one image. Pseudo-color was used to evidence all changes in fluorescence (scale shown on the right). Green arrows point to the time of DNA sample application and fuchsia arrows indicate the time of microarray reconstitution (Wash). For simplicity in this particular experiment the microarray was reconstituted twice, even though we have accomplished near to 10 times reutilization of the same microarray. (**C**) Time courses showing changes in mean fluorescence of each spot as lines. First and second sample application are indicated with green arrows.

Figure 2(A) illustrates the time course of MB1 fluorescence intensity increment in response to target addition (*pag*, protective antigen, gene sequence from anthrax) at room temperature. As illustrated in the figure, multiple replicate samples could be monitored simultaneously showing a robust increment in fluorescence upon target application (Figure 2(A,B), and supplementary video 1). Plotting x-y fluorescence against time provided in a single picture the entire history of the experiment (Figure 1(B)). This technique facilitates the visualization of the entire time course in a single image, and therefore will be extensively used in following figures.

Furthermore, the target could be dissociated and washed out from the microarray using the rinsing solution, resulting in the reconstitution of the microarray (Figure 2(B,C) and supplementary video 1). Using this procedure multiple sample applications on the same microarray could be performed (Figure 2(B), for simplicity only two repetitions are shown). Measuring fluorescence intensity over time provides target-MB association kinetics, which was reproducible among different applications (Figure 2(C)).

By using two MB designed to identify different regions from the *pag* (protective antigen) gene sequence from anthrax (Table 1, Experimental Section) we illustrate the identification of independent DNA signatures in targets (phenomenon named multiplexing). Multiplexing is extremely important to reduce the probability of false positives responses in microarray studies. Printing both molecular beacons (MB1 and MB2) on the same TIRF microarray in replicates shows the selectivity of this technique (supplementary video 1). Each MB produced fluorescence signal only when the respective complimentary oligonucleotide target was injected into the closed flow cell. Using a scramble DNA sequence (Material and Methods) did not affect MB fluorescence, illustrating the selectivity of the molecular beacons, something that was demonstrated since the first MB report [25].

Using different target concentrations provided the detection limit of the system. According to our results this method, using the particular configuration of LG-TIRFM-MB system described in this article, can detect targets in the picoMolar range (supplementary material Figure S2). One of the key factors in detecting low concentrations is the use of a very sensitive EM-CCD camera, and a powerful light source for excitation to produce a robust EW.

### 3.3. Measuring Realtime Association Kinetics in DNA-Protein Combined Microarrays

**Figure 3.**
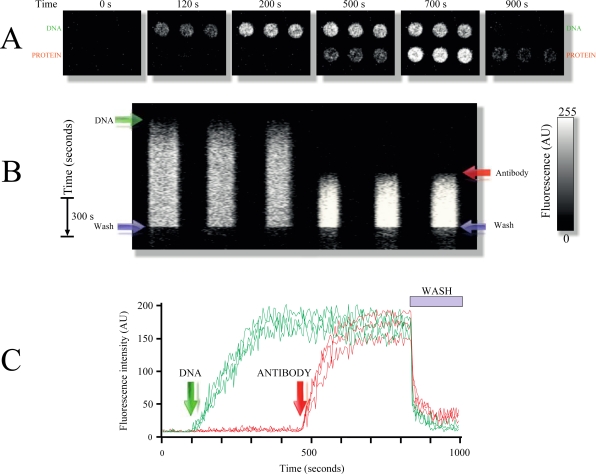
Combined DNA and protein microarrays on the same slide. (**A**) Example of a typical response obtained in a combined microarray using DNA and antibodies. The three spots at the top are molecular beacons for *pag* and the three bottom spots of Immunopure™ biotinylated mouse anti-human IgG. (**B**) Time course of the microarray response to bioanalyte solutions. First DNA from *pag* was applied (green arrow), followed by the secondary antibody (Alexa Fluor 532-labeled goat anti-mouse IgG, red arrow). Notice that even though the secondary antibody floods the entire chamber, only the spots containing the primary antibody produced a fluorescent signal. This simple experiment demonstrates the power of surface selectivity obtained with LG-TIRFM-MB. Microarray reconstitution brings the fluorescence towards baseline (pre stimulation) levels. At the right of the figure is shown the fluorescence intensity scale. (**C**) Lines depict time courses showing changes in mean fluorescence of each spot over time. Time points where DNA and antibody were applied are indicated by the arrows.

Figure 3 demonstrates an example of the use of DNA-protein combined microarrays. In this particular slide, spots were printed with MB1 to detect gene *pag* from anthrax, while other spots were printed with a primary antibody 1 used as a model protein for detection (Experimental Section).

To identify the antibody printed on the surface of the slide, a secondary antibody conjugated to the fluorophore Alexa 532 was utilized. Most interestingly, despite having the entire chamber bathed with the secondary fluorescent antibody, no background fluorescence was detected; only the fluorescence arising from the spot containing the first antibody was detected (antigen, Figure 3(B)). This experiment demonstrates the power of surface selectivity obtained with LG-TIRF-MB, which ensures that the bulk solution is not excited by the EW (the solution containing the secondary antibody), effectively reducing background fluorescence.

As illustrated in Figure 3(C), the combined DNA-protein TIRF microarray can be regenerated also using the same rinsing solution (Experimental Section). Using multiple replicate spots of the same probes demonstrates the robustness and reproducibility of the response in the TIRF microarray platform (Figure (3) and supplemental video 2).

Furthermore, we printed slides using two different primary antibodies to explore the possibility of identifying independent proteins in the same microarray slide. As illustrated in supplementary video 3, using different secondary antibodies we could identify different antigens (primary antibodies) printed on the TIRF slide in the same experiment. Most interestingly, we used the same fluorophore on both secondary antibodies (Alexa 532), but were able to identify the positive signal based on position of the different spots on the microarray slide. Because the excitation wavelength can be easily switched in the LG-TIRF-MB, one can envision more complex experiments utilizing multiple fluorophores with different excitation and emission spectra to identify simultaneously independent DNA and proteins present in samples of interest.

### 3.4. Association Kinetics Allows the Discrimination of Single Nucleotide Mismatches

One important feature on any POCD system is the ability to identify single nucleotide polymorphisms or mutations [26]. We were interested in exploring the selectivity limits of LG-TIRF-MB technology and to do so we designed experiments aimed to identify single and multiple nucleotide mismatches on the target via association kinetic analysis. In order to explore this, we introduced one or two mismatches in the *pag* (protective antigen) gene sequence from Anthrax (Material and Methods) and use these oligonucleotides with the MB1 molecular beacon (Experimental Section).

**Figure 4.**
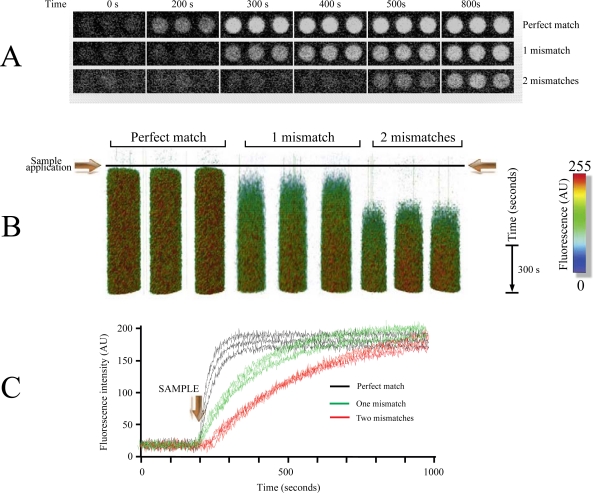
Identifying mismatches based on association kinetics from the micorarray. (**A**) Images obtained at different time points illustrating increments in fluorescence upon addition of three samples (DNA for *pag*). Samples are: perfect match to MB1, sample with one mismatch and sample with two mismatches (see Experimemtal Section for sequences). (**B**) Tridimensional rendering plot illustrating the entire microarray experiment time course in one image. This is a volumetric image plotting the changes in fluorescence overtime of each spot in 3D (x–y *versus* time). Pseudo-color was utilized to enhance even the smallest increment in fluorescence, making it easier to identify. The triplicate spots for perfect match, one and two mismatches are illustrated. (**C**) Time courses showing changes in mean fluorescence of each spot as lines overtime for perfect match (black lines), one mismatch (green lines) and two mismatches (red lines). Each line represents the mean fluorescence of one spot over time. Notice that the more mismatches present, the slower the increment in fluorescence.

In Figure 4(B) we show that using a 3D rendering technique facilitates visualizing the entire time course of the experiment in all the microarray spots in three dimensions (x-y and time). Combining the 3D rendering technique with pseudo-color provides a clear picture of the differences in the time courses for the association of the target to the different MB utilized in this experiment.

As illustrated in Figure 4(C), using LG-TIRF-MB technology was relatively simple to identify single and multiple mismatches by comparing target-MB association kinetics. This simple experiment demonstrates the flexibility and usefulness of measuring kinetics in microarrays.

Another important part of this new technology is the ability to print internal controls on the microarray. By doing this it is possible to normalize fluorescence response and mitigate for variations of excitation intensity, minimize false positives and also use internal controls to produce semi-quantitative results based on the fluorescence intensity obtained on each spot from the array.

## 4. Conclusions

Microarray technology has been present for the last fifteen years since the first developments of DNA in arrays for expression profiling described in 1995 [27]. Since those initial studies there have been several improvements and development of new systems for microarray technology.

New developments include the use of molecular beacons [28] and the design of protein microarray technology (for a review see [29]). Recent studies have shown the development of TIRF-based systems for microarray studies using microscopic beads [30]. Other new developments include the design of fiber optic microarrays [5] and the combination of microarrays with micro-fluidics [31].

The use of microarrays in medicine has been gaining attention in the last few years. Several studies highlight the advantage of microarray technology in pathogen detection and biodefense (for review see [13,32]), molecular diagnostics [15], single nucleotide polymorphisms (SNP) and mutation analysis [33], and cancer [34], among many others. Several of these studies point to the need of robust microarray technologies for POCD.

In the present study we report the development of a novel TIRF-based microarray technology for the identification of nucleic acids and proteins in the same microarray slide. For this purpose we utilize molecular beacons in combination with antibodies to print at low density on conventional microarray glass slides.

We used this combined DNA-protein microarrays in conjunction with a novel total internal reflection fluorescence system to integrate the microarray technology. This technology provides association and dissociation kinetics between target molecules and probes, which is useful in the discrimination of mismatches or to quantify the affinity of antibodies to antigens. By using internal controls with samples of known concentrations it is feasible to produce quantitative results.

Worth special mention, the technology described here can be easily extended for the identification of low molecular weight organic metabolites. In preliminary studies we have printed fluorescent assays for detection of cAMP, tryptophan and biotin. Similar strategies could be implemented for the identification of recognition elements for lipids, sugars or other metabolites.

Most importantly, this novel technology requires minimum sample preparation and no sample labeling, making it ideal for POCD. Furthermore, results from the microarray experiment are obtained in a few seconds, unlike conventional microarray technology, which requires several hours [35]. The speed is achieved by using TIRF technology to read the entire microarray simultaneously using a CCD camera, unlike conventional microarray readers which use confocal microscopy or scanners to read point-by-point the entire microarray [35].

Conventional microarray readers have lower single-to-noise ratios when compared to LG-TIRF-MB because confocal microscopy has a penetration depth of about 1 micron, whereas TIRF excites only a narrow area (approximately 100 nm) above the surface glass [22]. Any autofluorescence arising from bulk solution is discarded with LG-TIRF-MB (as clearly illustrated in Figure 3).

There are also limitations related to the use of LG-TIRF-MB. Firstly, because the system produces a wide EW (approximately 16 mm wide) all the optical power is diluted in a relatively large area (which is required to excite the entire microarray at once), therefore relatively powerful excitation light source is required (typically of 250 mW or more), to reach the detection limit determined by photostability of organic fluorophores. This limitation is significantly reduced in trough-objective TIRF (o-TIRF) systems where all the optical power is concentrated in a few microns. However, because the critical angle in o-TIRF can only be achieved using high numeric aperture/high magnification objectives, o-TIRF technology cannot be used for microarray studies where low magnification objectives are required. The decrease in intensity of the EW along the TIRF slide does not affect significantly the results, since only a small (less than 2%) reduction in EW intensity are typically observed at the end of the slide/lightguide [23].

Because in LG-TIRF-MB there is no scanning of the microarray pixel-by-pixel unlike in conventional microarray readers, this technology is limited to the viewing area attained by the objective. Using a low magnification objective approximately 60–100 spots can be visualized at once (depending on the spot diameter). This low-density microarray contrasts with the thousands of spots scanned on conventional high-density microarray platforms. Thus LG-TIRF-MB may be more appropriate for POCD or applications where exploring entire transcriptomes (or thousands of probes) is not required.

The limit of detection of LG-TIRF-MB for DNA and RNA targets is larger than that for PCR-based methods, yet the capability of simultaneous detecting DNA, RNA, protein, and metabolite markers (a task we can conduct routinely with LG-TIRF-MB) represents significant advantages of LG-TIRF-MB over traditional methods. Recent developments in PCR using CCD and in-gel technologies to analyze the results provide extended advantages to conventional PCR [36], yet detecting only DNA and RNA markers remains the major limitation of PCR-based techniques. The other advantage of LG-TIRF-MB when compared to PCR-based methods is that results are obtained in seconds, whereas PCR methods may require hours.

Finally, use of a very sensitive camera might be necessary to identify targets present at low concentration in samples. In theory, LG-TIRF-MB can detect single molecule fluorescence; however this level of sensitivity can be achieved only using low light EM-CCD cameras, like the one used in the present study.

## Supplementary Material



## Figures and Tables

**Table 1.** Illustrates the sequences of the complementary oligonucleotides and the sequences of molecular beacons used in the present study. For molecular beacons the sequence underlined identifies the stem, and the double underlined the mistmatch introduced. HEX refers to the fluorophore and BHQ1 (Black Hole Quencher 1) to the quencher.

**Probe**	**Sequence**
MB1 xo1pag (stem sequence underlined)	5′-(5HEX) CGG GTA CAG ATG CTA TAA TCA AAG TTC GTG TCC ATT(Biotin-dT)AC CCG-3′
MB2 xo1pag (stem sequence underlined)	5′-(5HEX) CGG GTA CTA ATC TAT GCC TAG TTC ATA TAC(Biotin-dT)AC CCG-3′
MB1-target complimentary DNA (Stem-complimentary sequence underlined)	5′-TTT GAC ATC AAC CGT ATA TCC TTC TAC CCG-3′
MB2-target complimentary DNA	5′-GAT TAG ATA CGG ATC AAG TAT ATG-3′
1-base mismatched DNA (mismatch in double underline)	5′-TTT GAC ATC AAC CC͇T ATA TCC TTC TAC CCG-3′
2-base mismatched DNA (mismatches show double underline)	5′-TTT GAC ATC AAC CC͇T AA͇A TCC TTC TAC CCG-3′
Non-complimentary DNA (Scrambled sequence)	5′-GAC TAT GCA GGT GCC GCG CGG AAA GGA TTT-3′
