# Supplementary material for: A Platform for Combined DNA and Protein Microarrays Based on Total Internal Reflection Fluorescence
Source: Sensors (Basel). 2012 Feb 9;12(2):1800–15. doi: 10.3390/s120201800 (PMC3304140; doi:10.3390/s120201800)

Supplementary Material

A Platform for Combined DNA and Protein Microarrays Based on Total Internal Reflection Fluorescence

Alexander Asanov [1,*], Angélica Zepeda [2,*] and Luis Vaca [3]

[1] TIRF Technologies, 951 Aviation Parkway, Suite 700, Morrisville, NC 27560, USA
[2] Instituto de Investigaciones Biomédicas, Universidad Nacional Autónoma de México, Ciudad Universitaria, DF 04510, México
[3] Instituto de Fisiología Celular, Universidad Nacional Autónoma de México, Ciudad Universitaria, DF 04510, México; E-Mail: lvaca@ifc.unam.mx

* Authors to whom correspondence should be addressed; E-Mails: asn@tirftechnologies.com (A.A.); azepeda@biomedicas.unam.mx (A.Z.); Tel.: +525-5622-9215 (A.Z.); Fax: +525-5622-9182 (A.Z.).

Received: 9 December 2011; in revised form: 13 January 2012 / Accepted: 2 February 2012 / Published: 9 February 2012

Abstract: We have developed a novel microarray technology based on total internal reflection fluorescence (TIRF) in combination with DNA and protein bioassays immobilized at the TIRF surface. Unlike conventional microarrays that exhibit reduced signal-to-background ratio, require several stages of incubation, rinsing and stringency control, and measure only end-point results, our TIRF microarray technology provides several orders of magnitude better signal-to-background ratio, performs analysis rapidly in one step, and measures the entire course of association and dissociation kinetics between target DNA and protein molecules and the bioassays. In many practical cases detection of only DNA or protein markers alone does not provide the necessary accuracy for diagnosing a disease or detecting a pathogen. Here we describe TIRF microarrays that detect DNA and protein markers simultaneously, which reduces the probabilities of false responses. Supersensitive and multiplexed TIRF DNA and protein microarray technology may provide a platform for accurate diagnosis or enhanced research studies. Our TIRF microarray system can be mounted on upright or inverted microscopes or interfaced directly with CCD cameras equipped with a single objective, facilitating the development of portable devices. As proof-of-concept we applied TIRF microarrays for detecting molecular markers from *Bacillus anthracis*, the pathogen responsible for anthrax.

Keywords: TIRF; molecular beacon (MB); point-of-care diagnostics (POCD); protein microarrays; DNA microarrays

---

Figure S1. Diagram illustrating the mechanisms for fluorescence initiation upon nucleic acids or protein binding to the probes on the TIRF slide. The cartoon-like diagram illustrates the hairpin structure of the molecular beacon in the absence of target DNA (left of the figure). In this structure both 3' and 5' ends of the probe are found in closed proximity, resulting in the quenching of fluorescence by the Black Hole Quencher (BHQ1). Upon DNA target addition the association of the beacon to the target (right of the figure) results in the separation of the fluorophore and the BHQ1 and the concomitant production of fluorescence signal. In the case of protein arrays, the second protein/antibody is labeled with a fluorophore, thus resulting in a fluorescence signal upon association to the first antibody attached to the surface of the TIRF microarray slide. In this type of configuration, each dot from the microarray (shown in the photograph at the bottom of the figure) represents either a DNA or a protein probe printed on the TIRF slide.

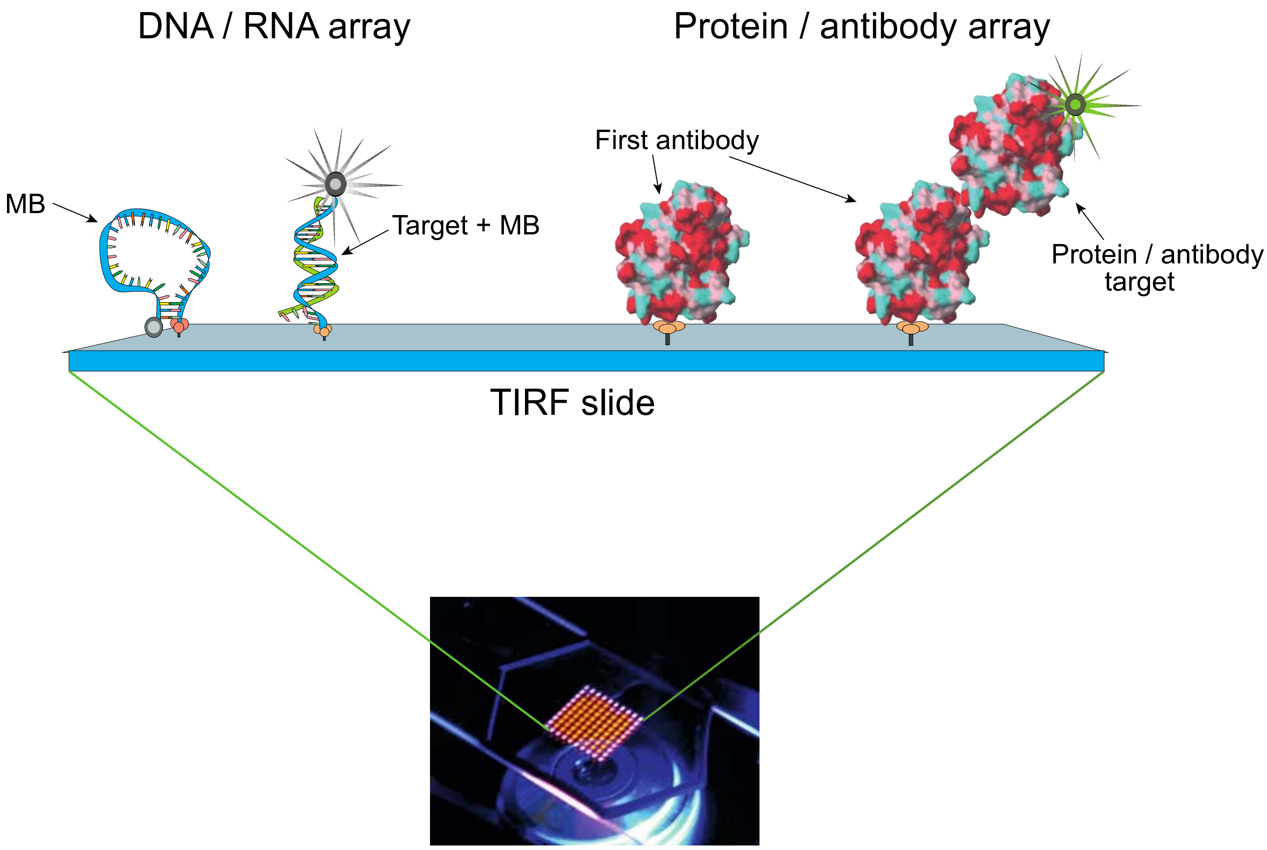

Figure S2. Sensitivity limit for DNA detection on a TIRF microarray. (**A**) Ten consecutive microarray spots replicates illustrates the reproducibility and sensitivity of our method when applying increasing concentrations of the *pag* gene (0, 0.1, 1, 10 and 100 nM). Red circle around the spots indicate the area utilized to measure the integral of fluorescence over time utilized to plot the graph shown in B. (**B**) Time course of fluorescence increments upon *pag* gene addition, graphs show that there is no difference between 100 and 1,000 nM of the sample, indicating that the system is saturated. (**C**) Mean and standard deviations of the increments in fluorescence intensity upon bioanalyte addition obtained from at least three independent experiments measuring 10 replicates.

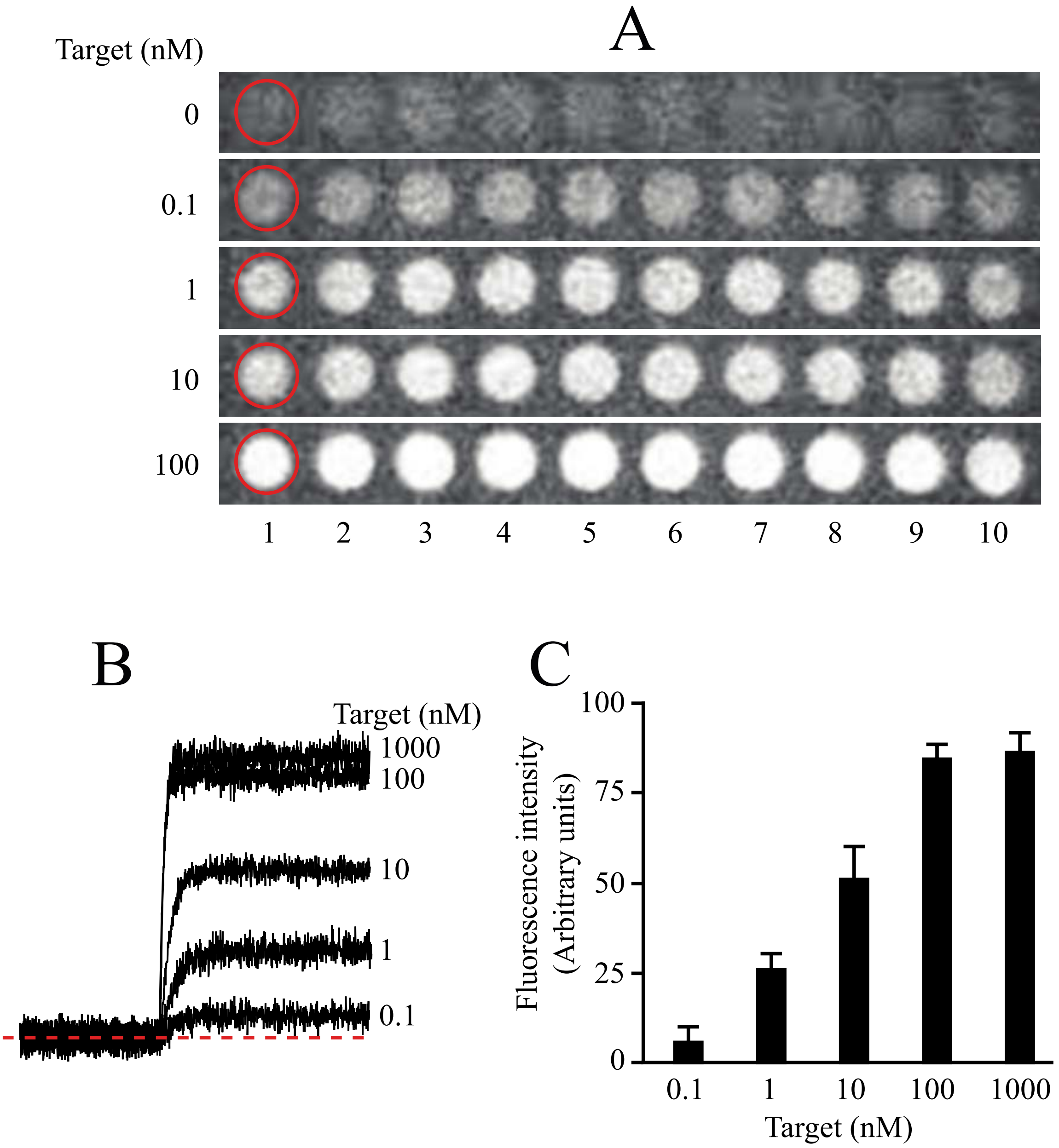

Supplement: Supplementary file 1 [file sensors-12-01800-s001.pdf]
